# A combined protocol for isolation, culture, and patch-clamp recording of dorsal root ganglion neurons

**DOI:** 10.52601/bpr.2024.240036

**Published:** 2025-04-30

**Authors:** Ruolin Wang, Yu Lu, Jianbo Zhao, Xueting Duan, Yang Chen, Zhuoyu Zhang, Rong Huang

**Affiliations:** 1 Neuroscience Research Center, Key Laboratory of Biomedical Information Engineering of Ministry of Education, School of Life Science and Technology, Xi’an Jiaotong University, Xi’an 710000, China; 2 Neurological Department of Tongji Hospital, School of Medicine, Tongji University, Shanghai 200333, China

**Keywords:** Sensory dorsal root ganglion neurons, Isolation, Culture, Patch-clamp recording

## Abstract

The dorsal root ganglion (DRG) neurons are crucial in transmitting sensory information from the peripheral nervous system to the central nervous system, including touch, pain, temperature, and proprioception. Understanding the functions and mechanisms of DRG neurons is essential for studying sensory processing and developing efficient treatments for sensory disorders. In addition, electrophysiological patch-clamp recording is a powerful and classical tool to study the functions and mechanisms of the nervous system. Building upon the strategies outlined in published works and our group’s abundant research experience in DRG neurons’ functions by patch-clamp, we have summarized and put forward a comprehensive step-by-step protocol combining juvenile rat DRG neuron isolation and culture, and patch-clamp recording. This protocol would be a powerful guidance document for neuroscience researchers to study sensory DRG neurons’ physiological and pathological functions using electrophysiological tools.

## INTRODUCTION

The dorsal root ganglion (DRG) neuron is a crucial component of the peripheral nervous system, and is responsible for detecting and transmitting sensory information to the central nervous system (Devor 1999; Meltzer *et al*. 2021). Located near the spinal cord, the DRG neuron is unique in its structure and function as the pseudounipolar neuron. Each DRG neuron has two axonal projections, one that extends to the peripheral nerve endings and another that connects to the spinal cord (Ramer *et al*. 2000). DRG neurons are critical in perceiving and responding to environmental stimuli, making them a key target for neuroscience research. Understanding the functions of DRG neurons is essential for developing efficient treatments for conditions such as chronic pain, sensory disorders, and nerve injuries (Berta *et al*. 2017).

Electrophysiological patch-clamp recording is a powerful technique (Carmeliet 2019; Verkhratsky and Parpura 2014) used in neuroscience and cell biology to study the activity of individual cells and the signal communication between neurons, providing valuable insights into ion channel functions, synaptic transmission, and cell signaling processes in various physiological and pathological conditions. By analyzing individual cells’ electrical properties, researchers can better understand the intricate mechanisms underlying cellular functions and dysfunctions. Although patch-clamp has been invented for over 40 years (Fenwick *et al*. 1982; Penner and Neher 1989), it is still a powerful and widely used tool in neuroscience research. Learning to do patch-clamp is essential to study the physiological and pathological functions and mechanisms of the nervous system.

Previous studies have introduced the protocol of DRG isolation and culture (Perner and Sokol 2021; Sleigh *et al*. 2016; Smith *et al*. 2023), as well as the patch-clamp recording (Chen *et al*. 2017; Gandini *et al*. 2014; Leyrer-Jackson *et al*. 2019; Lhomme and Prevot 2023). However, DRG isolation and culture are based on the adult mouse or rat, while little is known about newborn juvenile rodents. For patch-clamp recording, the description of using the operating software, like the PatchMaster here, and what the signal looks like on the software window for each step are still lacking. Therefore, it is necessary to provide a combined protocol of DRG isolation, culture, and patch-clamp recording, which would bring great convenience for junior researchers interested in neurobiology of sensory system, as well as the whole nervous system.

Our lab focuses on the physiological functions of sensory DRG neurons by patch-clamp recording, including the ion channel currents, the action potentials, the membrane capacitance reflecting neurotransmission, and the post-synaptic currents. Our previous work reported a new form of vesicle release mode which is Ca^2+^ independent but voltage-dependent in the soma of DRG neurons (Zhang and Zhou 2002) and the following work revealed its molecular mechanism (Chai *et al*. 2017) and further proved its presence in synaptic transmission (Wang *et al*. 2022). In addition, we also revealed the regulating mechanism of vesicle exocytosis coupled endocytosis in DRG neurons (Chen *et al*. 2022; Wang *et al*. 2016). Collectively, we have abundant experience in DRG neuron isolation, culture, and the corresponding patch-clamp recording (Huang *et al*. 2019; Zhang and Huang 2024). Building upon the strategies outlined in published works and our group’s abundant research experience in DRG neurons’ functions by patch-clamp, we have summarized and put forward a comprehensive step-by-step protocol with a combination of DRG neuron isolation and culture, and patch-clamp recording, which is much improved when compared with previous reports. This protocol would be a powerful guidance document for neuroscience researchers to study sensory DRG neurons’ physiological and pathological functions using electrophysiological tools.

## STEP-BY-STEP PROCEDURE

### Step 1: Preparation for DRG isolation

Step 1.1: Prepare the enzyme solution, including 1 mg/mL collagenase and 0.2 mg/mL trypsin. Dissolve them in DMEM/F12 solution and filter.

**[TIP]** 1.5 mL of enzyme solution is enough for 1–2 rat/mouse’s DRG tissues. The enzyme solution can be preserved at –20 °C before use.

Step 1.2: Incubate the coverslip glasses with 0.1 mg/mL Poly-L-lysine.

**[TIP]** Poly-L-lysine helps the neurons adhere better. Coverslip glasses are pretreated with concentrated sulfuric acid, prewashed with ddH_2_O, and preserved in 100% alcohol.

**Figure 1:**
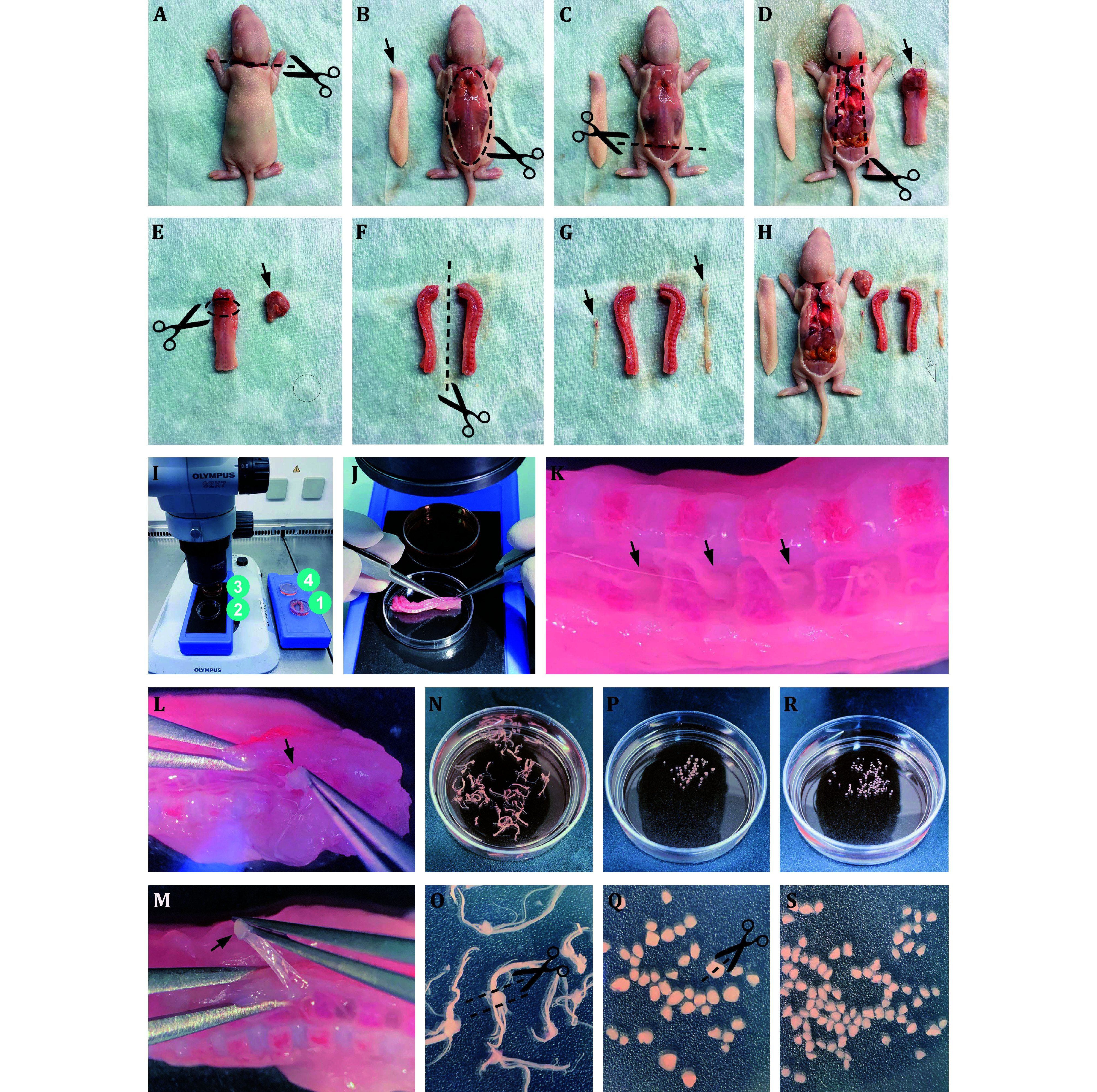
Images show each step of isolating DRG neurons from postnatal 5-day Sprague-Dawley rats. See more details in Step 2

Step 1.3: Prepare four 3.5 cm dishes and put them on the ice box, as shown in Fig. 1I.

Step 1.4: Add the appropriate amount of ice-cold L15 solution to three of the above dishes.

**[TIP]** It is recommended to isolate the neurons in the ice-cold solution. DMEM/F12 solution also works.

Step 1.5: Prepare the scissors and tweezers, and sterilize with 75% alcohol.

Step 1.6: Prepare a postnatal 5-day Sprague-Dawley rat, and anaesthetize it with ice block.

### Step 2: Introduction of DRG isolation in detail

Step 2.1: Prepare a simple dissection table with absorbent paper to absorb the blood.

Step 2.2: Put the anesthetized rat on the dissection table and sterilize it with 75% alcohol.

**[TIP]** Use a 75% alcohol watering can to spray all the surfaces of the dissection table and the animal. Sterilizing the dissection table and the rat with 75% alcohol completely would avoid bacteria contamination.

Step 2.3: Cut the cervical vertebra with scissors (Fig. 1A). Drain the blood.

Step 2.4: Cut off the skin of the back (Fig. 1B).

Step 2.5: Cut the lumbar vertebra (Fig. 1C).

Step 2.6: Cut the bilateral rib and take out the spinal column (Fig. 1D).

Step 2.7: Trim away the attached tissue on the cervical part of the spine (Fig. 1E).

Step 2.8: Split the spinal column in half (Fig. 1F).

**[TIP]** Pay attention to the direction of spinal column splitting. Evenly half-splitting is convenient for subsequent pinching out the dorsal root ganglions.

Step 2.9: Remove the spinal cord tissue (Figs. 1G and 1H).

Step 2.10: Put the above processed spinal columns into dish #1 (Fig. 1I).

Step 2.11: Take out the half spinal column and put it in dish #2. Pinch out the dorsal root ganglion one by one and put them in dish #3 (Figs. 1J–1N).

**[TIP]** The ganglions are located between two vertebrae (Fig. 1K). Each ganglion has two branches of nerve fibers (Fig. 1O), one is outside the intervertebral foramen while the other is buried inside the intervertebral foramen. Grab the nerve branch buried inside the intervertebral foramen, not the ganglion, with tweezers to avoid any damage to the ganglion (Figs. 1L and 1M).

Step 2.12: Cut off the fibers on both sides of the ganglion (Figs. 1N and 1O) and transfer the processed clean ganglion into dish #4.

Step 2.13: Cut each ganglion evenly in half (Figs. 1P and 1Q).

**[TIP]** Take the smallest ganglion as the final size standard and all ganglion pieces should be about the same size (Figs. 1R and 1S), to ensure that each ganglion piece receives the same degree of enzyme digestion.

Step 2.14: Discard the L15 solution and add the enzyme solution from Step 1.1.

Step 2.15: Put the above tissue-enzymes-containing dish into a 37 °C 5% CO_2_ cell incubator and wait for 40 minutes.

**[TIP]** During the 40 minutes of waiting, wash the coverslip glasses in Step 1.2 with ddH_2_O and wipe clean the surgical instruments carefully. Clean the dissection table and put the animal carcass into the freezer.

Step 2.16: At the 30^th^ minute, take out the ice-cold solutions of DMEM/F12 and DMEM/F12 with 5% Fetal Bovine Serum in advance. Prepare two 15 mL centrifuge tubes.

Step 2.17: At the 40^th^ minute, take out the dish in Step 2.15 and add 5 mL ice-cold DMEM/F12 solution to dilute and stop enzyme activity.

Step 2.18: Carefully suck out the ganglion pieces and transfer them to a new 15 mL centrifuge tube. Add 1 mL ice-cold DMEM/F12 solution.

Step 2.19: Blow the tissue carefully eight times using a 1 mL pipette.

Step 2.20: Carefully suck out the remained ganglion pieces and transfer them to another new 15 mL centrifuge tube. Add 1 mL ice-cold DMEM/F12 solution.

Step 2.21: Blow the tissue carefully eight times again using a 1 mL pipette.

**[TIP]** If some ganglion tissues remain, repeat Steps 2.20–2.21.

Step 2.22: Collect all single cell-containing suspensions in one 15 mL centrifuge tube. Make up the volume to 7 mL with ice-cold DMEM/F12 solution.

Step 2.23: Centrifuge at low speed, about 600 r/min for five minutes.

**[TIP]** 7 mL solution and low-speed centrifugation could help to get clean cell sediment. Impurities and cell debris will be retained in the upper liquid.

Step 2.24: Discard the supernatant and add the appropriate amount of DMEM/F12 with 5% Fetal Bovine Serum. Blow the cell sediment into cell suspension again and drop the cell suspension onto the coverslip glass surface pretreated with Poly-L-lysine.

Step 2.25: Put the above coverslip glass-containing 3.5 cm dishes into a 37 °C 5% CO_2_ cell incubator and wait for 20 minutes.

**Figure 2:**
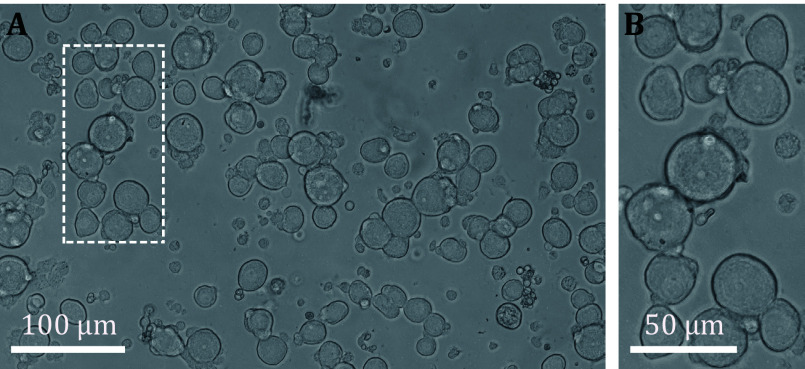
Images show acutely isolated DRG neurons (after ~2 h culture). **A** Acutely isolated DRG neurons. **B** An enlarged image from Panel A

Step 2.26: Take out the above neurons preparation and add 2 mL DMEM/F12 with 5% Fetal Bovine Serum for each 3.5 cm dish. Put them into a 37 °C 5% CO_2_ cell incubator again for culture (Fig.2) before use.

**[TIP]** For the newborn juvenile mouse DRG isolation, the protocol is almost the same as the above rat protocol. Because newborn juvenile mouse DRG-ganglions are much smaller than the newborn juvenile rat DRG-ganglions, there are some slight differences in isolating. In Step 2.13, the newborn juvenile mouse DRG-ganglions don’t need to be cut in half except the L4–6 big ganglions. In Step 2.15, the enzyme digestion time could be decreased to about 30–35 minutes.

### Step 3: Introduction of DRG culture

Step 3.1: On Day 1, use DMEM/F12 solution with 5% Fetal Bovine Serum for culture.

Step 3.2: For Day 2 and long-term culture, use Neurobasal solution supplemented with 2% B27, 1% GlutaMAX, and 10 ng/mL nerve growth factor.

**[TIP]** For long-term culture, the glial cell inhibitor of 5 μmol/L cytosine arabinoside should be added to the culture medium. Every two days, the culture medium should be replaced with a fresh new medium.

**[TIP]** For the newborn mouse DRG culture, the protocol is the same as the above rat protocol.

### Step 4: Preparation for patch-clamp

Step 4.1: Prepare the standard external Ca^2+^-containing solution, standard external Ca^2+^-free solution, and standard intracellular electrode solution. The formulas are listed in the Reagents part.

Step 4.2: Prepare the glass electrodes. It is bought from WPI (World Precision Instruments Inc.), with the specification of 1.5 mm × 8 cm.

**Figure 3:**
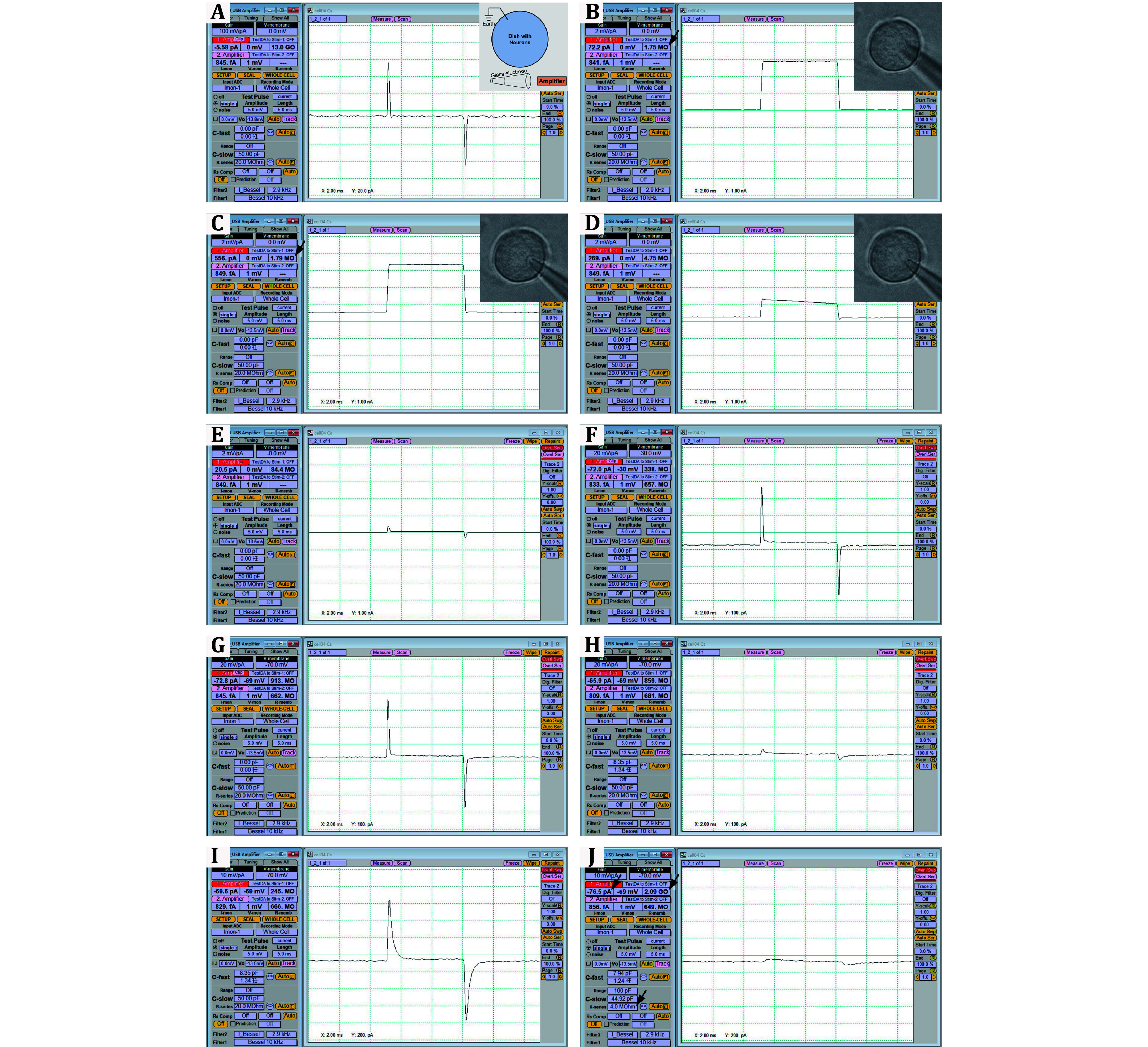
Images show the PatchMaster software windows of each step of the whole-cell patch-clamp recording mode. The insert in Panel A is a schematic diagram of the patch-clamp setup. The inserts in Panels B–D are photographs showing the relative position of a patch-clamp electrode and the DRG neuron. See more details in Step 5

Step 4.3: Use a glass microelectrode puller to get suitable electrodes that can be used for patch-clamp recording. The electrode resistance is about 2 MΩ (Fig. 3B).

**[TIP]** The bigger electrode resistance means a smaller electrode aperture, making it harder to break the membrane for whole-cell mode patch-clamp recording. The smaller electrode resistance means a bigger electrode aperture, making it harder to get a GΩ seal (Fig. 3J) for patch-clamp recording.

### Step 5: Introduction of patch-clamp in detail

Step 5.1: Place a coverslip glass with adherent DRG neurons into the recording chamber filled with standard external Ca^2+^-containing solution.

Step 5.2: Screen and choose a good DRG neuron.

**[TIP]** This step is important. The good DRG neurons should adhere to the glass coverslip well and have a clean and intact membrane with no holes. Independent neurons are better than clustered neurons. The regenerated axons are a good marker reflecting the healthy and active condition of the cultured DRG neurons. In addition, we prefer to choose cells with small or medium diameters in our experiments, which standard could be changed to adapt for the experiments with specific purposes.

Step 5.3: Fill the tip of the recording glass electrode with the standard intracellular electrode solution. Put the electrode into the holder of the amplifier probe.

Step 5.4: Before putting the electrode into the standard external Ca^2+^-containing solution, apply a positive pressure to the electrode. Two reversed wave peaks can be seen in the PatchMaster software window (Fig. 3A).

Step 5.5: Put the electrode into the solution and click the “SETUP” button. Check if a square wave appears in the PatchMaster software window (Fig. 3B).

**[TIP]** In this step, before touching the DRG neuron, the electrode resistance (as shown in Fig. 3B) can reflect whether the glass electrode is blocked or broken. If the electrode resistance is not right after the electrode has been put into the solution, immediately prepare a new glass electrode.

Step 5.6: Use a micromanipulator to move the glass electrode directly above the cell (Fig. 3B) and gently touch the cell until a pit is formed (Fig. 3C). After touch, the resistance should have a slight rise (from 1.75 to 1.79 MΩ in Fig. 3C or bigger).

**[TIP]** How to move the glass electrode to the target neuron quickly is a critical step, which needs a lot of training.

Step 5.7: Release the positive pressure (Fig. 3D) in Step 5.4 and apply a suction pressure gently (Fig. 3E).

**[TIP]** In this step, it would be good if the square wave changes to a horizontal line (Fig. 3E). Meanwhile, the resistance should rise steadily (from 4.75 to 84.4 MΩ, Figs. 3D and 3E).

Step 5.8: Apply negative command voltage from 0 mV to –70 mV with a step amplitude of 10 mV (Figs. 3F and 3G). The Gain of the *Y* axis could be adjusted according to the signal appearance (Figs. 3E and 3F).

**[TIP]** In this step, when applying command voltage, check the leakage current incessantly (from +20.5 to –72.8 pA, Figs. 3E–3G). It would be better if the leakage current is within –100 pA.

Step 5.9: Click the “C-fast Auto” button in the software window (Fig. 3H). The two reversed wave peaks should decrease to small peaks.

Step 5.10: Use a skillful suction pressure to break the membrane to form whole-cell recording mode (Fig. 3I). The two reversed small wave peaks should increase to big peaks suddenly.

**[TIP]** For junior learners, it is effective to click the “Zap” button in the software to break the membrane. The suggested “Zap” amplitude is 500 mV, and the duration is 0.2 ms.

Step 5.11: Click the “C-slow Auto” button in the software (Fig. 3J). The two reversed wave peaks should decrease to small peaks again.

**[TIP]** Till now, the whole-cell recording mode has been successfully got. Check the leakage current, resistance, and R-series (Fig. 3J). For DRG neurons, the leakage current should be within –200 pA (smaller is better), resistance should be about 1 GΩ (higher is better) and R-series should be about 3–10 MΩ. For the HEK293 cell line, the leakage current should be within –50 pA.

**Figure 4:**
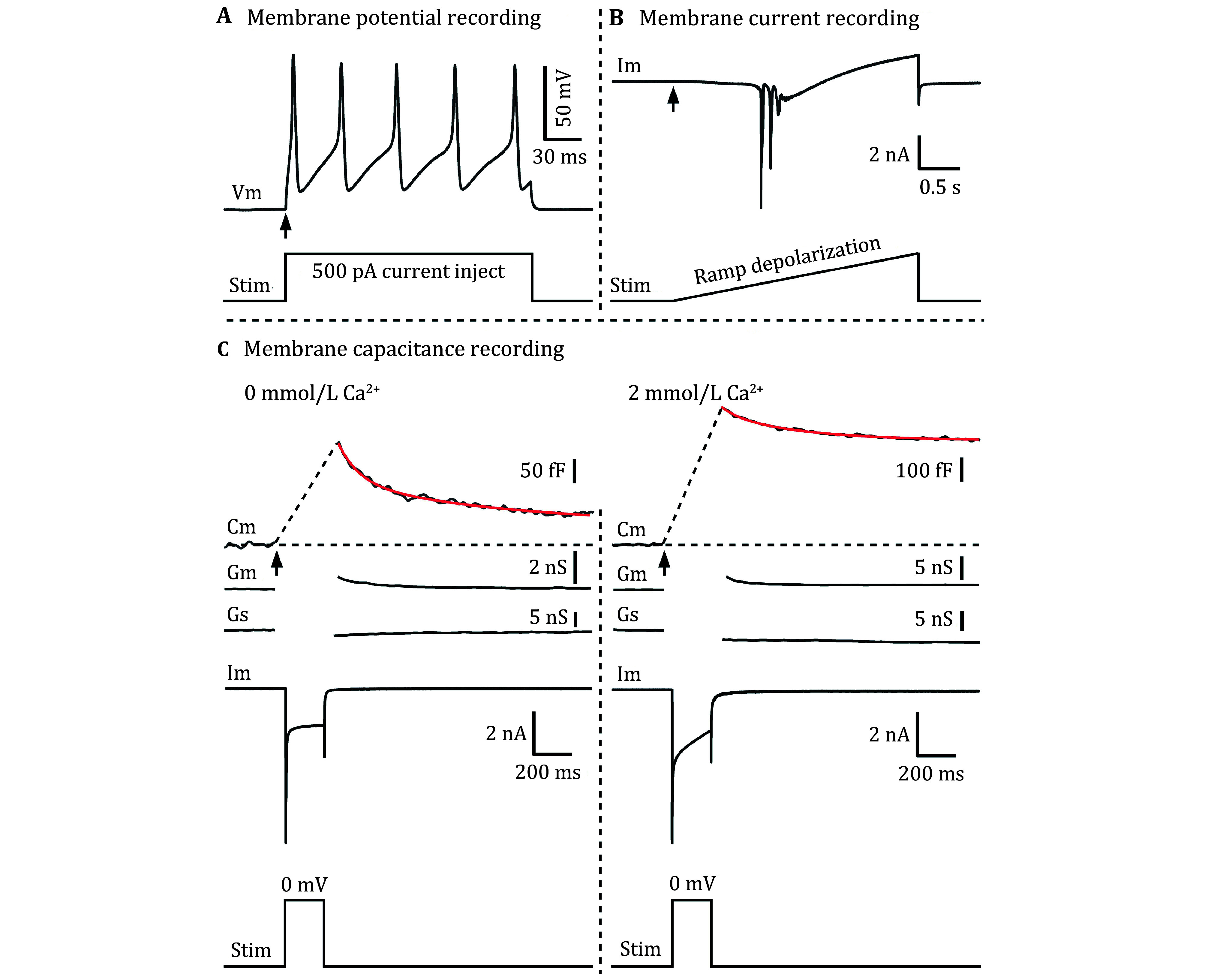
The action potentials, currents, and membrane capacitance signals from acutely isolated DRG neurons. **A** Membrane potential (Vm) signals (Upper), evoked by 500 pA current injection (Lower protocol, stim). **B** Membrane current (Im) signals (Upper), evoked by ramp depolarization (Lower protocol, stim). **C** Membrane capacitance (Cm, Upper) signals, evoked by 200 ms depolarization from –70 mV to 0 mV (Lower protocol, stim), from Ca^2+^-free solution (Left) and 2 mmol/L Ca^2+^-containing solution (Right), respectively. The Gm and Gs reflect the quality of capacitance recording, and Im is the current signal (Middle)

Step 5.12: Select or design different protocols and record the corresponding electrophysiological signals, such as action potentials, ion currents, and membrane capacitance (Fig. 4). The solution puff-system is needed to switch/add different solutions/drugs, like the Ca^2+^-free external solution, and different drugs experiments.

Step 5.13: Save the file.

### Step 6: Introduction of electrophysiological signals analysis offline

Step 6.1: The igor software is adopted for the offline analysis. Raw data is loaded through “PPT→Load Pulse/PM file→File→Open→Do it”.

Step 6.2: The raw data can/should be smoothed through “Analysis→Smooth→Algorithm→Smoothing→Do it” to obtain a better signal-to-noise ratio for display.

**[TIP]** For membrane capacitance data, the 50 ms trace after the stimulus-ends should be deleted as this 50 ms signal cannot reflect the real vesicle release signal.

Step 6.3: Click the button of “Windows→New Layout→Select Objects→Do it” to make figures.

Step 6.4: Click the button of “File→Save Graphics→Do it” to export figures.

Step 6.5: Click the button of “File→Save Experiment” to save the original document for further analysis.

## ADVANTAGES AND LIMITATIONS OF THIS PROTOCOL

This step-by-step procedure provides a standardized guideline to isolate and culture the primary sensory dorsal root ganglion neurons and perform the patch-clamp recording on the sensory neurons. As skillful operators, we provide many tips for each important step, which can help the junior learners handle the key points of isolation, culture, and patch-clamp better. However, we should admit that this step-by-step procedure still lacks some essential steps for patch-clamp, like how to build an electrophysiological platform from scratch, eliminate electrical noise, and analyze the electrophysiological signals with more introductions. Therefore, it is more suitable for the learners with basic electrophysiological knowledge. In addition, depending on different research purposes, this protocol may need slight adjustments to align with specific demands, like modifying from newborn rat to adult rat or from rat to mouse. With the ongoing progress in electrophysiological research on neural activity and the continuous advancement of techniques, the protocol for studying the physiological functions and underlying mechanisms of sensory neurons needs to be expanded and updated.

## MATERIALS AND EQUIPMENT

### Reagents

• L15 (Sigma, Cat. No. L4386)

• DMEM/F12 (Thermofisher, Cat. No. 12400024)

• Fetal Bovine Serum (Thermofisher, Cat. No. 10099141C)

• Collagenase (Sigma, Cat. No. 9001-12-1)

• Trypsin (Sigma, Cat. No. 9002-07-7)

• Poly-L-lysine (Sigma, Cat. No. 25988-63-0)

• Neurobasal (Sigma, Cat. No. 21103049)

• B27 (Sigma, Cat. No. 17504044)

• GlutaMAX (Sigma, Cat. No. 35050061)

• Cytosine Arabinoside (Selleck, Cat. No. S1648)

• Standard external Ca^2+^-containing solution (in mmol/L): 150 NaCl, 5 KCl, 2.5 CaCl_2_, 1 MgCl_2_, 10 H-HEPES, and 10 D-glucose, pH 7.4

• Standard external Ca^2+^-free solution (in mmol/L): 150 NaCl, 5 KCl, 1 EGTA, 1 MgCl_2_, 10 H-HEPES, and 10 D-glucose, pH 7.4

• Standard intracellular electrode solution (in mmol/L): 153 CsCl, 1 MgCl_2_, 10 H-HEPES, and 4 Mg-ATP, pH 7.2

### Equipment

• Amplifier, EPC 10 USB Double, from HEKA, http://www.heka.com/index.html

• Micromanipulator, CFT8301E, from RUIQI, https://xuzhou062512.11467.com

• Microscope, IX73 inverted microscope system, from OLYMPUS

• Microscope, SZX7, from OLYMPUS

• Glass Microelectrode Puller, PC10, from NARISHIGE

• Scissors, S12003-09, from RWD

• Scissors, 54110BDM, from 66VT

• Scissors, 54137B, from 66VT

• Tweezers, 500233, from WPI

• Tweezers, FC12001T-11, from RWD

### Software and algorithms

• PatchMaster: from HEKA, http://www.heka.com/index.html

• Igor: from WaveMetrics, https://www.wavemetrics.com/

## Conflict of interest

Ruolin Wang, Yu Lu, Jianbo Zhao, Xueting Duan, Yang Chen, Zhuoyu Zhang and Rong Huang declare that they have no conflict of interest.
